# Artificial Intelligence and Machine Learning Methods to Evaluate Cardiotoxicity following the Adverse Outcome Pathway Frameworks

**DOI:** 10.3390/toxics12010087

**Published:** 2024-01-19

**Authors:** Edoardo Luca Viganò, Davide Ballabio, Alessandra Roncaglioni

**Affiliations:** 1 Laboratory of Environmental Toxicology and Chemistry, Department of Environmental Health Sciences, Istituto di Ricerche Farmacologiche Mario Negri IRCSS, 20156 Milan, Italy; alessandra.roncaglioni@marionegri.it; 2 Milano Chemometrics and QSAR Research Group, Department of Earth and Environmental Sciences, University of Milano-Bicocca, 20126 Milan, Italy; davide.ballabio@unimib.it

**Keywords:** in silico models, artificial intelligence, machine learning, new-approach methodologies (NAMs), toxicological endpoints, quantitative structure–activity relationship (QSAR), adverse outcome pathway (AOP)

## Abstract

Cardiovascular disease is a leading global cause of mortality. The potential cardiotoxic effects of chemicals from different classes, such as environmental contaminants, pesticides, and drugs can significantly contribute to effects on health. The same chemical can induce cardiotoxicity in different ways, following various Adverse Outcome Pathways (AOPs). In addition, the potential synergistic effects between chemicals further complicate the issue. In silico methods have become essential for tackling the problem from different perspectives, reducing the need for traditional in vivo testing, and saving valuable resources in terms of time and money. Artificial intelligence (AI) and machine learning (ML) are among today’s advanced approaches for evaluating chemical hazards. They can serve, for instance, as a first-tier component of Integrated Approaches to Testing and Assessment (IATA). This study employed ML and AI to assess interactions between chemicals and specific biological targets within the AOP networks for cardiotoxicity, starting with molecular initiating events (MIEs) and progressing through key events (KEs). We explored methods to encode chemical information in a suitable way for ML and AI. We started with commonly used approaches in Quantitative Structure–Activity Relationship (QSAR) methods, such as molecular descriptors and different types of fingerprint. We then increased the complexity of encoders, incorporating graph-based methods, auto-encoders, and character embeddings employed in neural language processing. We also developed a multimodal neural network architecture, capable of considering the complementary nature of different chemical representations simultaneously. The potential of this approach, compared to more conventional architectures designed to handle a single encoder, becomes apparent when the amount of data increases.

## 1. Introduction

Cardiovascular disease is a multifactorial condition involving a combination of genetic, environmental, and lifestyle factors. There are many different types of cardiovascular disease, each with its own set of risk factors and mechanisms for development. Accurately predicting the onset or progression of cardiovascular disease in humans can therefore be very difficult. In silico methods are essential for addressing these issues and reducing the need for traditional in vivo testing.

The battery of computational tools is fundamental to a thorough understanding of the impact of chemical compounds on cardiac tissues, considering both the hazard and the exposure to compounds, and serves as a first-tier component for more complex evaluation architectures such as new-approach methodologies (NAMs) or next-generation risk assessment (NGRA) [1,2]. The results from different in silico methods can always be used, coupled with in vitro and omics results, in weight-of-evidence strategies. This means that possible toxic chemicals can be given priority for further testing, with the aim of examining only some specific compounds in vivo [3]. It would be ideal to extend current modeling capabilities, since most work published nowadays dealing with the potential cardiotoxicity hazard of chemicals focuses primarily on drugs and their potential impact on hERG channel inhibition [4,5,6,7]. However, hERG channel inhibition is only one of the possible interactions between chemicals and biological targets that could lead to cardiotoxic effects. Additionally, drugs make up just one class of compounds capable of inducing these effects [8], while many others may significantly contribute to these effects, considering that cardiovascular diseases are among the leading causes of mortality worldwide [8]. This is particularly noteworthy, especially considering that the evaluation of potential cardiotoxic effects of chemicals, such as industrializers, pesticides, biocides, and mixtures thereof, is still limited, poorly addressed, and poorly regulated.

We focused on modeling the potential hazards of compounds following the concept of Quantitative Structure–Activity Relationships (QSARs) [9,10]. QSAR is a computational method employed to predict a potential compound’s biological activity by considering its chemical structure and other relevant properties, and models based on this approach analyze how the properties of a molecule are related to its activity. Recent advances in artificial intelligence (AI) and machine learning (ML) have revolutionized predictive toxicology, holding promise for enhancing the safety assessment of different classes of chemicals. These methods are particularly well suited for our purposes. AI and ML can rapidly analyze large datasets, identify patterns, and learn from experience, thus improving the accuracy of predictions. Then, we aim to demonstrate the potential of AI-based QSAR models to predict cardiotoxicity using well-defined endpoints as biological targets defined by the theory of the Adverse Outcome Pathway (AOP). This allows the development of conceptual frameworks used in toxicology and risk assessment to describe and understand the sequence of events linking a molecular initiating event (MIE) to an adverse outcome such as increased mortality from heart failure. 

In accordance with the events outlined in the linear AOP framework for cardiotoxicity (https://aopwiki.org/aops/480, accessed on 18 November 2023), we collected data from biological assays providing information on the potential toxic interactions between chemicals and specific biological targets. The data came from various sources, and depending on the amount and quality of the compounds tested, we employed different ML and AI architectures. One fundamental topic in these approaches is extracting and using the chemical information hidden in a molecule identifier, such as chemical names or the Chemical Abstracts Service (CAS) numbers that can be found in datasets, in a way suitable for ML and AI. 

There are various methods to present chemical information, and we employed the most widely used ones to provide the best description for each biological target. This is a crucial step towards achieving high modeling performance since different sources of information can describe specific toxicity mechanisms more effectively than others. 

We explored different methods to encode chemical information in a suitable format for ML and AI. Starting with commonly used approaches in QSAR strategies, such as molecular descriptors and various types of fingerprints, we gradually increased the complexity of encoders. This involved incorporating methods based on graphs, auto-encoders, and character embeddings commonly used in neural language processing. This facilitates a full comparison of methods for capturing chemical information. 

In addition, our efforts were not limited to identifying the optimal encoder for each target; we tailored the AI architectures to handle multiple chemical encoders simultaneously, employing a multimodal approach. This enables our model to learn from diverse chemical representations and gain insights from various perspectives on the same compounds, making for greater predictive efficiency. 

The key objective of a multimodal neural network is to leverage the complementary nature of the different modalities to improve overall performance and gain a fuller understanding of the data. By combining information from multiple sources, the network can capture richer patterns, correlations, and context that may not be evident when analyzing each modality in isolation [11]. The potential of this approach compared to more conventional architectures designed to handle a single encoder becomes apparent when the amount of data increases. 

Cardiotoxicity assessment has often been investigated by modeling the mechanisms most often targeted by drugs molecules (for instance, hERG inhibition) [5,6,12], but other mechanisms like mitochondrial dysfunction appeared to be relevant especially for environmental contaminants [8]. At the same time, mitochondrial dysfunction is a mechanism of toxicity common to other Adverse Outcome Pathways targeting other endpoints (e.g., liver toxicity); therefore, several works have been published in the literature that partially address the same topic [13,14]. Various methods, such as multitask or ensemble methods, are explored to cover endpoints like mitochondrial dysfunction toxicity. In our cases, we have developed different AI and ML approaches. These approaches utilize and compare different chemical representations to evaluate which one is best suited for each specific case. This involves considering a broader spectrum of molecular initiating events (MIEs) and key events (KEs) with the ultimate goal of achieving a more comprehensive understanding of the biological toxicity interaction that leads to cardiotoxic effects. This understanding is a fundamental first step in building a battery of computational tools aimed at evaluating the impact of chemical compounds on cardiac tissues.

## 2. Materials and Methods

### 2.1. Datasets

We collected data from the ICE database (https://ice.ntp.niehs.nih.gov/, accessed on 15 October 2023), which provides high-quality curated data to support the development and evaluation of new, revised, and alternative methods. ICE contains datasets curated by NICEATM, ICCVAM, and their partners to meet specific quality standards and are useful in evaluating or developing new approaches for assessing chemical safety. We selected the datasets related to cardiotoxicity, specifically on the mode of action called Cardiomyocyte/Myocardial Injury. The data obtained in this manner can be presented as a matrix, each row corresponding to a different chemical, and the columns showing the biological assays in which the compounds are tested. The chemicals retrieved are univocally defined by the CAS number, a unique numerical identifier assigned to each chemical described in the scientific literature. We then retrieved the Simplified Molecular Input Line Entry System (SMILES) from CAS using in-house software (https://github.com/EdoardoVigano/Chemical-Resolver, accessed on 15 October 2023). We represent the compounds using SMILES because it is a widely used and standardized notation system for representing chemical structures and molecules, using text strings. Atoms and bonds are represented by alphanumeric characters, with specific rules for various chemical features, such as double bonds, rings, and branches. This representation of chemicals is compact and human-readable, making it useful for computer-based chemical informatics and chemical databases. 

We curated the data on the retrieved SMILES. This involved standard SMILES canonization, followed by the removal of structures displaying inconsistencies that might indicate chemical errors and any duplicate structures. We also excluded stereochemistry and removed salts, concentrating solely on the largest fragments. This approach to SMILES curation is very commonly used [15].

The total number of curated chemical SMILES was then grouped based on the types of assay used to examine the compounds. This results in a series of specific datasets, one for each of our endpoints, namely the MIE and KE highlighted by the AOP frameworks [16]. An MIE is the first interaction of a stressor with a biological system at the molecular level. This interaction can be highly specific, such as chemical binding to a particular protein complex, DNA, or receptor, or non-specific, such as when reactive chemicals cause modifications of proteins. The KEs are biological or chemical events or changes in an organism that are vital in the pathway leading to an adverse outcome [16,17].

**Figure 1:**
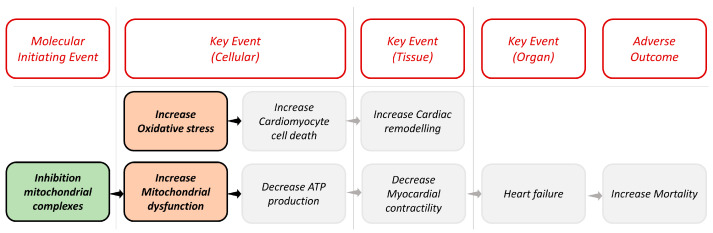
Representation of the linear AOP framework for cardiotoxicity. The colored blocks in the image are the biological targets considered in our work, and the gray ones are other potential events that could lead to heart failure.

In our case, following the linear AOP for cardiotoxicity as shown in Figure 1, we considered the inhibition of mitochondrial complexes (MIE1) as MIE and the increase in oxidative stress (KE1) and the increase in mitochondrial dysfunction (KE2) as KEs. We selected specific biological assays that have a critical role in identifying potentially harmful compounds able to interact as stressors in these biological targets. Table 1 lists the assays used. All information about assays is available in the ICE database (https://ice.ntp.niehs.nih.gov/, accessed on 15 October 2023).

The data are labeled as active or inactive for classification modeling purposes. We define activity as follows: a chemical is considered active in a particular MIE or KE if it shows a hit call label as active in at least one of the selected assays for that specific biological target; otherwise, it is labeled as inactive. The individual assay label was available in the files downloaded from the ICE platform. In particular, raw data provided by a vendor or laboratory underwent processing, indexing, transformation, and normalization using standardized methods. Subsequently, the concentration–response data are subjected to modeling through three selected models (constant, Hill, and gain–loss). If any models fit sufficiently, the chemical–assay pair is considered ‘active’ (hit call = active); otherwise, the final hit call is ‘inactive’.

Characteristics of the data collected and class proportions are shown in Table 2. The datasets are unbalanced for all three endpoints, and the statistics on dataset composition are reported. That information is important to consider applying a method for oversampling the minority class and selecting the right metrics to assess the model’s performance.

### 2.2. Molecular Representation

**Figure 2:**
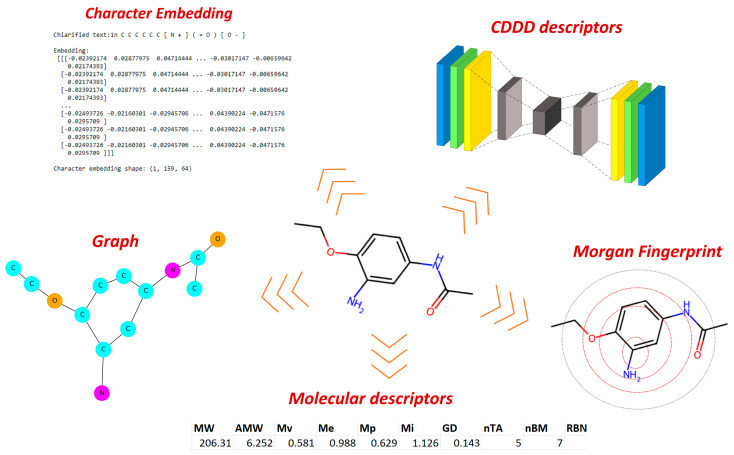
Chemical encoders used to extract chemical information from chemical identifiers suitable for AI and ML methods.

To encode chemical information, we adopted various approaches, beginning with the conventional methods typically employed in QSAR studies and gradually exploring more conceptually advanced techniques. Our initial steps involved molecular descriptors (MDs) [18], as well as different types of fingerprints like Morgan fingerprint and Molecular ACCess System (MACCS). Continuing this progression, we further extended the method by extracting chemical information through graphs and integrating NLP concepts, such as character embedding. Finally, we assessed the latent representations generated by the SeqToSeq encoder–decoder architecture as CDDD [19]. The types of encoders tested are summarized in Figure 2.

Not all these approaches to encode chemical information are suitable for each ML model or AI architecture since, in some cases, particular encoders require a specific AI architecture (all architecture used and specific encoders are reported in Supporting Information, Section S2). 

MMPN needs a specific architecture to manage the graph as input and must be capable of performing the message-passing process to update the vectors that describe the nodes’ representations, considering the neighborhood of the atom. This architecture is specific to managing graphs and cannot be used for other encoders, or vice versa. 

NLP architecture can process SMILES directly as text using specific layers for text vectorization and character embedding, which describe each character in SMILES as a numerical vector. Additionally, the layers used to handle this embedding typically involve managing sequences, such as Gated Recurrent Unit (GRU) and Long Short-Term Memory (LSTM). As a result, NLP architectures are designed specifically to handle SMILES as a sequence of characters, and no other encoder can be used as input in natural language processing (NLP). 

Regarding MD and fingerprints, the models use the same architecture, the only difference depending on the input dimensions, which are specific for each encoder.

Continuous and Data-Driven Descriptors (CDDDs) comprise latent representations generated by a complex encoder–decoder architecture trained on a massive number of SMILES, and this architecture can be separated from the one used for predictions. This approach allows us to treat CDDD as a descriptor calculator, providing a numerical vector for each SMILES, which can be used as input for deep neural networks, like what we have carried out with fingerprints and MD.

All the encoders and models, whether part of baseline ML, advanced deep neural networks, or other AI methods, were calculated and implemented using Python packages. The versions of the libraries and packages used are documented in Section 2.8.

#### 2.2.1. Molecular Descriptors

MDs enable us to describe each compound using thousands of numerical indices representing different chemical properties, such as polarizability, steric hindrance, molecule shape, etc. In other words, MDs are quantitative representations of chemicals, capturing various aspects of their chemical structure, properties, or behavior. We calculated the MDs using Mordred packages [20], obtaining 1613 different 1-2D descriptors.

#### 2.2.2. Fingerprints

Morgan fingerprints and MACCS binary fingerprints are used to encode chemical information. They are calculated using RDKit (version 2023.03.1) Python packages. Morgan fingerprints are a method for translating the structural information of a molecule into a fixed-length binary or bit-string presentation. This type of fingerprint does not rely on a predefined fragment library. Instead, they are generated by enumerating all possible fragments within a molecule, up to a certain size limit. The fragments are then converted into numerical values using a hash function. The MACCS fingerprint instead is a binary method that encodes the presence or absence of predefined structural fragments or substructures in a molecule. This fingerprint is designed to capture key chemical features of molecules, making it valuable for various tasks such as similarity searching, virtual screening, and structure–activity relationship (SAR) analysis.

#### 2.2.3. Molecular Graphs

When dealing with graphic representation, each compound is described as a graph where each atom is a node with a list of features, such as atomic number, valence number, number of hydrogen atoms bonded, and hybridization. Similarly, the chemical bonds are encoded as bonds in a graph with some features such as the bond type and a Boolean value indicating whether it is conjugated or not.

#### 2.2.4. Character/Word Embedding

Character/word embedding is applied directly to the SMILES string. Tokenization is used for this and involves breaking text into smaller units, called tokens. Tokenization is a fundamental step in NLP to analyze and process text. When splitting SMILES into characters, we found that 95% contained fewer than 111 characters. That is important when selecting the output sequence in the process of vectorization. For the text vectorization, we have to select the length of the output sequence and the vocabulary that contains all the different characters in the dataset plus a token to define the start and end of the sequence. The character vocabulary defined this way contained 28 different characters.

**Figure 3:**
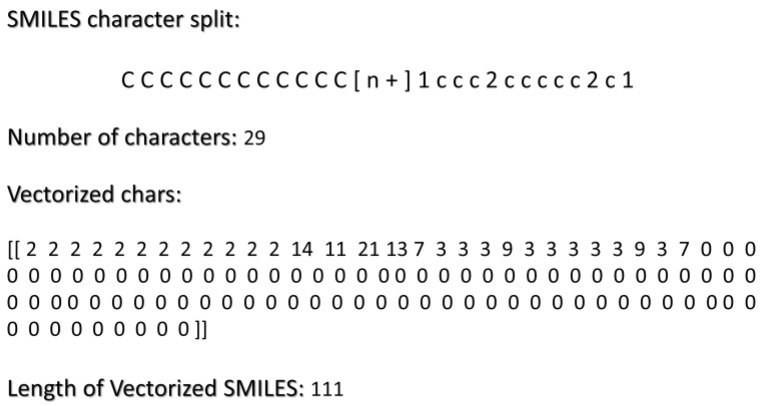
The image depicts the results of vectorization on SMILES, employing a vocabulary of 28 components and an output length of 111, the 95th percentile of SMILES length.

The results of these vectorization processes are illustrated in Figure 3.

The tokenized SMILES can be used as input in an embedding layer where each token is represented by a numerical vector in the network. The model learns this embedding during training and is the best way to describe the characters for our compound prediction objectives.

#### 2.2.5. CDDD: Latent Descriptors

Latent representation is used to describe the molecule in a very particular way that is based on the concept of encoder–decoder architecture [19], which is a fundamental component in AI and ML. This architecture is actually vital in tasks like natural language processing and image analysis. An encoder is responsible for transforming input data, such as text, SMILES strings, or images, into a compact and meaningful form called a latent representation. This captures essential information from the input data while reducing its size. This enables the auto-encoder to present complex chemical structures in a more manageable format. 

This latent representation can be used for various tasks like similarity analysis, clustering, and—as in our case—even for property prediction describing compounds for QSAR modeling. We used an architecture already trained on millions of chemicals [19]. CDDD is an auto-encoder often used for dimensionality reduction, which is particularly useful in chemistry to manage high-dimensional data.

### 2.3. Applicability Domain 

The applicability domain (AD) of a QSAR model describes the model’s constraints within its structural domain and response space. This validation principle limits the model’s applicability to accurately predict test samples that share structural similarities with the training samples used to construct the model. 

Over the years, various approaches have been proposed to define the AD of QSAR models. The differences among these approaches are mainly in the algorithms employed to characterize the AD within the descriptor space where the model can make reliable predictions [21,22]. 

To calculate the AD, we used the Applicability Domain Toolbox (for MATLAB) (Milano Chemometrics and QSAR Research Group) [23]. This tool implements a set of AD approaches based on several strategies, such as bounding box on PCs, convex hull, leverage, distance to centroid, k-nearest neighbors (kNN) approach with fixed k, k-nearest neighbors (kNN) approach with variable k, and probability-density-function-based methods. We defined the AD using methods such as range-based methods, geometric methods, and distance-based methods, specifically ‘Bounding box’, ‘Bounding box PCA’, ‘Leverage’, ‘Distance from centroid’, and ‘Distance kNN—variable k’ [24,25]. These methods are used to reach a consensus; therefore, if a chemical in the test set is defined as out of domain by all these algorithms, it is considered potentially to be discarded.

This evaluation was conducted for each encoder considering these approaches as a further consensus, so we discarded from our test set only the compounds that turned out to be out of the domain in any type of encoder. In other words, only compounds classified as out of domain in each AD method and for each chemical encoder were discarded. This decision reflects our aim to maintain a certain uniformity in the dataset without having different sets for each encoder, making for a more straightforward comparison. No data were out of domain, so no test data were discarded.

### 2.4. Model Architecture and Data Augmentation

We tested a wide range of different ML models and AI architectures to predict the potential toxic effects of chemicals on diverse events highlighted in AOP frameworks for cardiotoxicity. Selecting the model types and AI architecture depends on different factors such as the amount of data obtained and the type of encoder selected. 

We started with conceptually simpler and more common methods, gradually increasing the complexity of ML models, ending with a multimodal architecture that can consider different types of encoders simultaneously. We could only use these methods on KE2 because it is the only endpoint with enough data to train this type of architecture. A summary of the tests to establish the best model-encoder for each endpoint is reported in Supporting Materials Table S3. 

For all endpoints, including MIE1, KE1, and KE2, we selected a baseline battery of ML models, including logistic regression (LR), decision tree (DT), random forest (RF), balanced random forest (BRF), extreme gradient boosting (XGB), support vector machine (SVM), k-nearest neighbors (KNN), and Gaussian Naive Bayes (Gaussian NB). For the analysis of KE2, we gathered enough data to assess various deep learning approaches, including deep neural networks (DNNs) and message-passing neural networks (MPNNs), as well as architectures commonly used in NLP, that contain layers such as GRU and LSTM. We also explored multimodal architecture. 

Details of the architecture of each model are provided in Section S3 of the Supplementary Materials.

**Figure 4:**
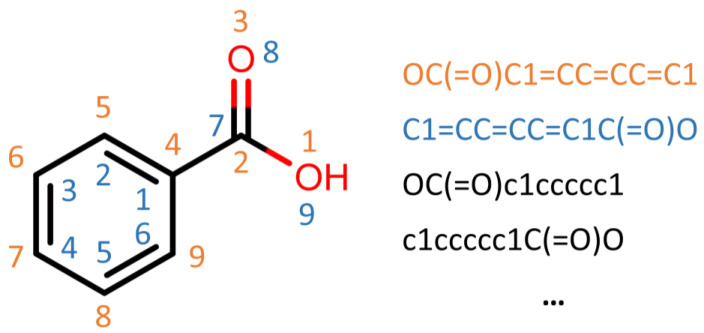
Example of how to achieve data augmentation on SMILES. The numbers represent the atomic enumeration based on the SMILES string, meaning the atom enumerated as ‘n’ is the ‘n’-th character in the string. The colors are used as an example to identify the different SMILES and the position of their atoms/characters.

For the NLP architecture, we employed a character embedding model with an architecture able to work directly on SMILES strings, encoding every single character as a numerical vector. This way, the networks learn the SMILES grammar and correlations between strings or sequences of characters and endpoints. With this approach, we can use data augmentation methods, writing the same SMILES in different way without changing the chemicals’ meaning, increasing the numbers in the training set by a factor of almost 10 (Figure 4). 

Data augmentation is an essential technique to increase the diversity and size of training data in ML, particularly when the data are limited. We used the SMILES enumeration approach [26]. Here, the fact that multiple SMILES represent the same molecule is explored as a technique to increase the data of a molecular QSAR dataset modeled by convolution layers and an LSTM cell-based neural network. 

The architecture used to manage the graph encoder using message-passing neural networks (MPNNs) is proposed in the literature [27]. These networks are particularly useful for tasks involving data with relational or graph-like structures, such as molecules, social networks, recommendation systems, and others. MPNNs operate on these molecular graphs through a series of message-passing iterations. During each iteration, nodes (atoms) exchange messages with their neighboring nodes (atoms), incorporating information about their local chemical environment that could be relevant to predict the potential activity of chemicals, finding a correlation between the local environment and the specific toxicity mechanism of action.

**Figure 5:**
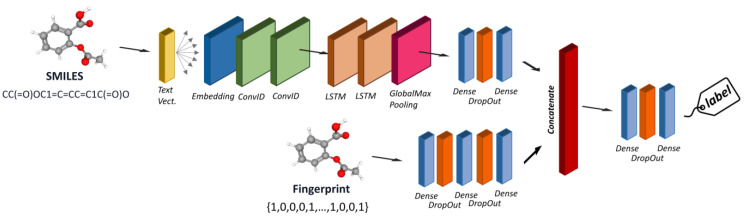
Architecture of a multimodal model able to learn from different chemical encoders.

The last architecture test was designed to consider different encoders and belong to the class of multimodal methods. An example is reported in Figure 5.

The architecture of a multimodal neural network typically consists of multiple branches, each dedicated to processing data from a specific modality. These branches can consist of various neural network layers, such as convolutional layers for images, recurrent or transformer layers for text, and fully connected layers for numerical data. The features learned by each branch are then fused or combined in a merging layer, where the model integrates the information from different modalities. We developed a multimodal architecture capable of simultaneously considering all the descriptors tested individually, except for the graph. This architecture includes five branches, each consisting of a deep neural network designed to handle one of the different descriptor types we used, such as MACCS, extended-connectivity fingerprints, MDs, SMILES as text, and CDDD. Regarding the architecture of each branch, the same principles apply to the individual models. For instance, the branch responsible for processing the SMILES string is based on an NLP architecture with text vectorization and embedding layers, followed by LSTM and GRU layers. The branches designed to handle numerical input, such as fingerprint or MD descriptors, consist of a series of dense layers with dropout. Joint training of all modules, with concatenation as a merging mechanism, was used to facilitate the convergence of the network.

### 2.5. Data Pre-Processing and Validation

Model validation is an essential step for producing high-quality models that can make accurate predictions that generalize well. A precise pipeline must be followed for model validation.

#### 2.5.1. Data Split

It is common practice to split the original dataset into training and testing sets. When dealing with datasets with a small number of compounds, such as MIE1 and KE1, we conducted a 90–10% training/set split, while for KE2, we tested different splits, progressively increasing the size of the training set, starting from 70–30% and ending with 90–10% (Table 3). This choice was made with the aim of evaluating the models’ behavior as the training dataset became bigger. Ideally, a good model should consistently perform better when the training set increases. For KE2, we could do these tests thanks to the amount of data available. In each split, we maintained the ratio between the toxic and non-toxic labels in the original dataset. The results for each split are reported in Supporting Information Tables S1 and S2. 

To train DNN efficiently, some of the training data (10%) are used in the validation set; this split is required to fine-tune the model’s parameters properly. 

#### 2.5.2. Data for Modeling for Each Encoder

The second step in the validation pipeline, after the data split, involves data pre-processing. This varies, and the method for preparing the data can differ significantly depending on the type of encoder. For instance, MD requires several distinct phases, including managing possible errors in MD calculations and scaling the resulting values. Scaling data in QSAR modeling is essential to ensure consistent and accurate model performance by addressing issues related to variable units and algorithm sensitivity. 

Other encoders are less demanding in the preparation phase. For instance, NLP models based on word embeddings can take canonical SMILES as input directly. MD data were standardized using standard scaling, which involves centering the variables around the mean and scaling to unit variance. Descriptors with a variance less than 0.1 or closely correlated descriptors (correlation exceeding 0.9) were discarded for CDDD descriptors and MD. For graphs, fingerprints, and word embeddings, no data curation was conducted in preparation for modeling.

The data preparation for deep neural networks and AI architectures differs slightly from traditional data processing. Besides handling data values, tasks such as checking for Not a Number [NaN] values and data scaling, one must also consider the computational cost and optimize the training time to create the best conditions for model convergence and stability during training. One way to achieve this is by normalizing activations within each mini-batch, which can reduce the likelihood of overfitting. 

In the case of our AI architecture, the dataset was divided into mini-batches of 32, and we employed TensorFlow for data prefetching. This technique is used to enhance the training performance of deep learning models by overlapping the data loading and model training phases. The goal of data prefetching is to minimize the idle time of the GPU or CPU during training and mitigate the impact of data loading latencies on overall training speed. By keeping the computational units, such as GPUs or CPUs, fully utilized, data prefetching achieves faster training times and more efficient model convergence. 

#### 2.5.3. Unbalanced Datasets

One of the most important limitations we encountered to reaching good performance for modeling is the presence of unbalanced classes. This is a very common problem with real data provided by biological assays, and data distribution makes it challenging for a model to learn and predict the minority class effectively. In addition, models operating on imbalanced data can seem very accurate when measured by traditional accuracy metrics, yet they may perform poorly in practice. 

To avoid these issues, we adopted different strategies commonly used in these situations. With baseline models belonging to ML methods, we employed a Synthetic Minority Over-sampling Technique (SMOTE) [28,29,30] on the training set. SMOTE works by generating synthetic samples of the minority class to increase its representation in the dataset. This is performed by selecting a minority class sample and then finding its nearest neighbors. A new synthetic sample is then created by randomly selecting a point between the minority class sample and one of its nearest neighbors. This process is repeated until the desired number of synthetic samples has been generated. This approach has different variants regarding the way to generate synthetic data, and we tested different ones such as K-means-SMOTE, SVM-SMOTE, and Borderline-SMOTE 1 and 2 [29,30]. The performance was best with SMOTE for MIE1 and KE2 and SMOTE-Borderline 1 for KE1. 

Additionally, we evaluated whether the synthetic data generated by the methods selected for the final model maintains an acceptable distribution compared to the original dataset. The results are reported in Section S5 of the Supporting Information. If the distribution difference between the augmented data and the original training dataset is substantial, the augmented data may not contribute effectively to model training. However, for each of our datasets, the difference between the original data and the dataset after the SMOTE approach remains acceptable. 

Regarding the architecture of deep learning, the methods to manage unbalanced datasets are different. During the training phase of DNN, the values of parameters are randomly selected by uniform distribution, and the models by backpropagation adjust these values, but initial guesses are not always the best. One can set the output layer’s bias to reflect the unbalanced distribution of data, and this can help with initial convergence. 

#### 2.5.4. Internal and External Validation

After pre-processing and oversampling, the data can be used to train and validate models with internal and external validation. For ML models, the internal validation involved 10 iterations of k-fold cross-validation, and during these iterations, we evaluated the internal performance of different ML methods and encoders to predict our endpoints. The results are reported in detail in Section S1 of the Supporting Information. Only the best combination encoder-model was further considered for the parameter tuning phase.

Regarding deep learning architecture such as multimodal models, MPNN, DNN, and NLP, the methods for internal evaluation are conceptually similar but different. The models are trained on the training dataset for multiple epochs. Each epoch comprises one forward pass and one backward pass for all the samples. During each epoch, input data are processed through the model to make predictions, and model parameters are updated based on the prediction errors evaluated on the validation set. This iterative process enables you to track the history of your selected optimization metric as it converges to a minimum over epochs.

For the external validation, we incorporated other metrics in addition to the F1-score and balance accuracy to show a fuller assessment of the model’s predictive abilities, so we could evaluate more deeply the model’s ability to generalize the knowledge gained from the training set. The metrics selected to evaluate the model’s performance on external tests are balance accuracy, precision, sensitivity, specificity, Matthews correlation coefficient (MCC), and F1-score.

Precision is the ratio of true positive predictions to the total number of positive predictions made by a model. Specificity measures the ability of a model to correctly identify negative instances out of all actual negatives. Sensitivity, also known as recall, measures the ability of a model to correctly identify positive instances out of all actual positives. The Matthews correlation coefficient is a metric that takes into account true positives, true negatives, false positives, and false negatives to provide a balanced measure of classification performance. F1-Score is the harmonic mean of precision and sensitivity (recall), and it provides a balance between precision and recall and is especially useful when there is an uneven class distribution.

A detailed explanation of the indices used to evaluate the models is provided in the Supporting Materials Section S3.

### 2.6. Tuning Parameters 

After initial screening to select the best combination encoder-model, we ran another five iterations of k-fold cross-validation to tune the hyperparameters, using grid-search. Grid search can systematically explore different hyperparameter combinations, allowing the selection of optimal settings that enhance the model’s predictive performance. The results for each baseline ML model are reported in Supporting Information Section S1. F1-score is the metric employed to evaluate the models during hyperparameter optimization because it is the most meaningful since it can be applied for unbalanced datasets. 

For DNN, we ran a grid search to explore the numbers of layers and nodes to use in the fully connected part of the architecture, but always considering the computational cost. Many hardware platforms, including CPUs and GPUs, are optimized for operations involving powers of 2. Therefore, selecting a number of nodes in a layer that has a power of 2 enhances the efficiency of architectures in terms of memory and computation. This optimization results in faster training and inference processes. During the training phases for DNN, to reduce the computational cost and optimize the hyperparameters, we set the checkpoint to monitor the convergence of the network. If there was not any improvement in validation loss for n-epochs, the learning rate parameter was decreased by a factor of 10, but if there was still no improvement, the networks ended the training phase to avoid overfitting.

### 2.7. Explainability Methods

In this work, we focused our efforts on achieving high model performance, sacrificing, in part, the model interpretation, at least for the more advanced approach. However, we examined the models obtained to provide insights about potentially relevant features associated with the toxic outcome. This was performed focusing on the models developed for the smaller datasets, namely MIE1 and KE1, where the best models obtained were based on ML methods, and the encoder was molecular descriptors and physico-chemical properties. Depending on the type of models and encoders, different approaches can also be used to perform Explainable Artificial Intelligence (XAI) analysis, such as SHAP or LIME [31,32]. SHAP facilitates global model interpretability by extending its analysis beyond individual predictions, providing a comprehensive view of feature importance through the consideration of average contributions across all predictions. In a few words, the Shapley value is the measure of the average marginal contribution of a feature.

Additionally, the permutation importance method was employed. Randomly re-ordering a single descriptor in the dataset should cause less accurate predictions, and the model performance will suffer especially when a descriptor that the model relied on heavily for predictions is shuffled.

### 2.8. Software

All models and architecture implementations were performed with Python packages. Python 3.9.16, RDKit (version 2023.03.1), scikit-learn 1.2.2, SciPy 1.8.1, imbalanced-learn 0.10.1, pandas 1.5.3, matplotlib 3.7.1, and deepchem 2.7.1 xgboost 1.7.5,libraries were used for ML implementation, oversampling methods, data analysis and data exploration, and data visualization. TensorFlow 2.12.0 and Keras 2.12.0 were used to create architecture for deep learning models as multimodal and NLP methods. The packages used for XAI are eli5 version 0.13.0 and SHAP version 0.44.0. 

## 3. Results

### 3.1. Baseline Models

In the initial phase of the modeling process, we focused on establishing a baseline model by testing the most commonly used ML methods. We specifically selected encoders suitable for these approaches, such as MDs, Morgan fingerprints, MACCS, and CDDD latent descriptors. We aimed to assess the best combination of model descriptors for each endpoint by systematically evaluating all possible combinations of model types, descriptors, and biological targets. The results for each model and descriptor are detailed in the Supplementary Materials, Sections S1 and S2. Table 4 reports the best results after a parameter grid search for hyper-parameters. The exploratory analysis, which involved internal validation, was used to identify the most effective combination of models and chemical encoders. Mordred molecular descriptors and latent descriptors from CDDD were the preferred methods for encoding chemical information for MIE1, KE1, and KE2. The model types that performed best are respectively k-nearest neighbors, logistic regression, and extreme gradient boosting.

The performance is quite satisfactory, especially when considering the limited amount of data available, particularly for KE1 and MIE1, where there are only a few hundred samples. One must also bear in mind that the in vitro tests used for chemical evaluation may give some false positives. False positives would have a significant impact on our dataset because we incorporate multiple assays, and our labeling criteria define a compound as ‘active’ if it gives positive results in just one assay. 

The presence of a certain number of false positives might also be attributable to the oversampling approach. While oversampling methods have proved fundamental in improving the performance of the models, they do have the side effect of increasing the rate of false positives.

### 3.2. Baseline Models XAI

We explore the importance of descriptors for the assessment provided by ML models, as they have advantages compared to more advanced AI approaches in being less complex and, therefore, more transparent.

For KE1, the selected models are of the logistic regression type, which is a very transparent method where the descriptor weights can be easily accessible. Access to model weights allows us to create a hierarchy of importance for the descriptors, and we can understand how these descriptors affect the assessment. Also, for KE1, the chosen encoder was molecular descriptors that have chemical-physical meaning, which could also be interpretable from a toxicological perspective. We found that for KE1, the ATS (autocorrelation of a topological structure) descriptor has high weight in logistic regression parameters. In these descriptors, the atoms of a molecule were represented by properties such as atomic mass or partial charge. The distance between atoms was measured as the number of bonds between the respective atoms (topological distance). Then, it could mean, from a toxicological perspective, that the shape of molecules and charge distribution has a fundamental role in chemicals’ ability to trigger the biological targets that affect the KE1 and that should be further explored. 

Regarding the MIE1 model that is KNN, we chose to explore the descriptors’ importance using the permutation importance method; the results are reported in Table 5.

These listed descriptors are defined in this way:

- MAXsOH: max. number of OH with a single bond.
- MAXdssC: max. number of C with double and two single bonds (=C<).
- GATS7d: Geary coefficient of lag 7 weighted by valence electrons.
- AATS5i: averaged Moreau–Broto autocorrelation of lag 5 weighted by ionization potential.
- AATS8se: averaged Moreau–Broto autocorrelation of lag 8 weighted by Sanderson EN.
- NssS: number of S with two single bonds.

This suggests that a specific feature in molecules, such as an alcohol group or double bonds, and more generally, a small region with high electron density, holds significance in the presence of the inhibition of mitochondrial complexes.

**Figure 6:**
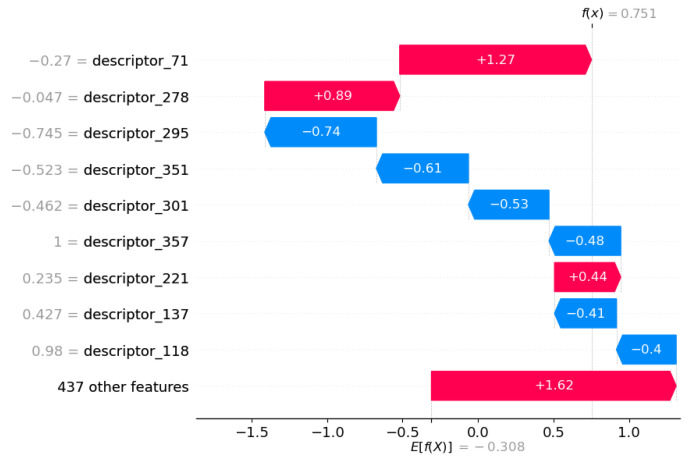
Features contributing to push the model output from the base value (the average model output over the training dataset we passed) to the model output. Features pushing the prediction towards higher values are shown in red; those pushing the prediction towards lower values are in blue. KE2 with CDDD as encoder.

For KE2, the models and encoders selected for higher performance exhibit less transparency compared to MIE and KE. Indeed, we chose XGB models trained on chemicals described by CDDD descriptors. This implies that, even though we can explore how descriptors can impact the assessment, the challenge lies in thoroughly understanding the specific CDDD descriptors that have been identified as highly important, particularly in terms of their implications for toxicology or chemical-physical meaning, as described in Section 2.2. Despite this interpretative limitation of CDDD, we decided to use the SHAP method to explore descriptor influence on decision making performed by the model. The results are reported in Figure 6.

The results suggest that there are important features related to semantic and grammar rules in SMILES notation that, when decoded by CDDD descriptors, become relevant to performing the chemical assessment. Further exploring these rules could be fundamental to finding a bridge between chemical property and toxicological effects regarding KE2.

### 3.3. Deep Neural Networks and AI Architectures

In the second part of our modeling work, we progressively increase the complexity of AI architecture to predict KE2 to outperform the optimized ML. We selected this biological target because the amount of data available allows us to use more advanced deep learning methods commonly used in other fields that require at least some thousands of training data. We built DNN architectures with multiple layers and ran many tests, examining the different types of activation functions, optimizers, learning rates, and numbers of layers and nodes to find the optimal in terms of results and computational cost. The models’ results are reported in Table 6, and details of the models’ architectures are reported in the Supporting Materials in Section S2.

We used the same architecture of DNN on different encoders for a similar evaluation of the ML models. In this case, for DNN, the CDDD latent descriptors seemed the most promising to capture the chemical information to predict the effects of chemical toxicity on the KE2 endpoint.

The results from both NLP and multimodal approaches are promising. With multimodal methods, we have developed an architecture capable of managing all the encoders we tested. Consequently, as the dataset grows bigger, and the training data becomes more extensive, these methods tend to outperform even the best baseline ML models.

### 3.4. Baseline Models Compared with Deep Neural Networks and AI Architectures and Encoders

**Figure 7:**
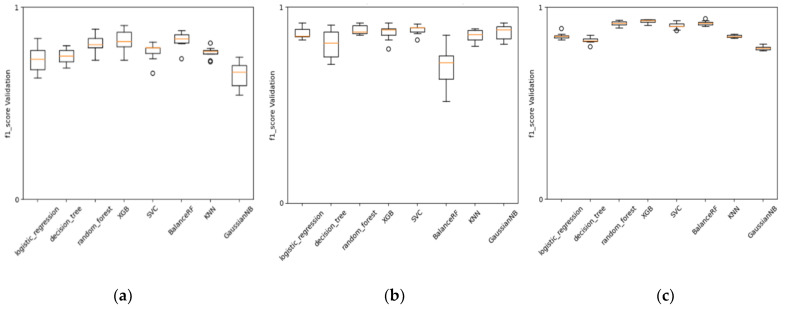
Comparison of models’ performance (F1-score) in ten-fold cross-validation. (**a**) KE1 with MD as encoder, (**b**) MIE1 with MD as encoder, and (**c**) KE2 with CDDD as encoder.

As mentioned earlier, we found the best encoder for MIE1 and KE1 involved Mordred molecular descriptors. The behavior of these descriptors during cross-validation tests, as shown in different types of ML baseline methods (Figure 7), demonstrates a certain level of stability in performance across various folds. The performance achieved with these descriptors is satisfactory and confirms the efficiency of a more classical approach in QSAR modeling using MDs, especially when the data are in the order of hundreds, and there are not enough data to permit a more advanced architecture that learns both the encoding and predictive assessment simultaneously (like, for instance, in NLP using character embedding as a layer).

The results for KE2 differ. In this case, we had enough data to adopt advanced AI architectures. Some of these, during training, can simultaneously learn to encode chemical information and improve their predictive efficiency. NLP with data augmentation performs best when the dataset is split into a 90–10% train–test ratio (BA 0.815). NLP with embedding layers updates the vectors that represent characters in a text sequence during the training phase. This means that the AI architecture is learning how to encode the information and how to use this information to assess chemicals at the same time. Therefore, the amount of data available becomes crucial, which explains why the data augmentation approach significantly boosts NLP performance, by 4.5%. This improvement starts from baseline accuracy (BA) of 0.780 for NLP and ends with a BA of 0.815 with data augmentation.

Another encoder we found very promising in almost all endpoints, giving good stability in performance using different models, is the CDDD latent descriptor. This turned out to be the best overall to build the baseline model for KE2. 

**Figure 8:**
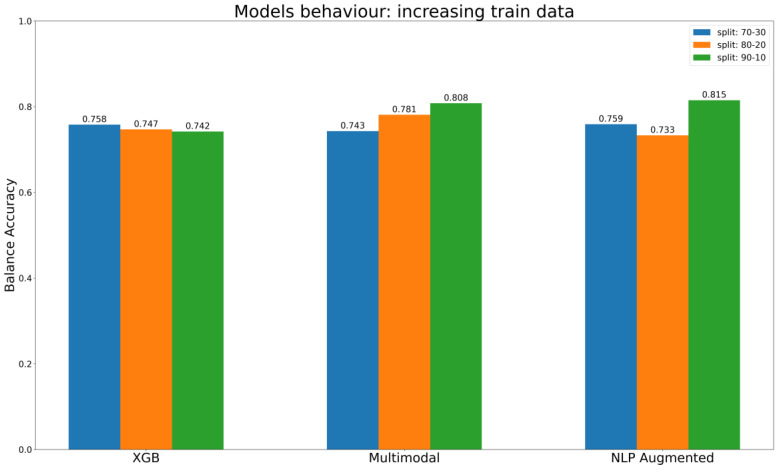
Comparison of models’ performance (balance accuracy) with progressive increases in the training dataset.

In summary, the results of our assessments of different methods for encoding chemical information are consistent with our expectations. Descriptors that rely on predefined methods for encoding information, such as MD, fingerprint, and CDDD pre-trained, have limitations in representing the full spectrum of information contained in a chemical structure. These methods are limited by their intrinsic properties, which prescribe a predefined way of describing the compound, and models trained on such representations are limited to only determining the correlation between the information encoded and the endpoint labels, without taking an active part in the extraction of useful information from chemical identifiers such as SMILES. This limitation is then reflected in the results of the models themselves, which reach a plateau in performance despite the increase in training data, as shown in Figure 8 regarding the best baseline ML models (XGB) to predict KE2. In other words, models detect correlations but are limited by the predefined representation of the chemical information. This important limitation is overcome in cases of AI architecture such as multimodal approaches, which use multiple parallel ways to encode chemical information, and NLP models, where the embedding layer is trained as part of the network (Figure 8). The evaluation described in Figure 8 used different splits, gradually increasing the size of the training set, to gain insights into the models’ behavior. The expectation is that the models will consistently improve their performance or, at the very least, maintain it as the training set gets bigger. It is interesting that the behavior of the multimodal approach aligns with this ideal scenario, its performance constantly increasing with the increase of data in the training set. The NLP models with data augmentation reach the best overall performance with the last split, in conditions with a maximum number of compounds in the training set. Ultimately, the XGB, which belongs to the baseline models, maintains a certain stability, but its performance does not increase as the training test gets bigger.

In general, with no further consideration about the encoder type, deep learning methods have proved competitive and often outperform baseline ML techniques, as is evident in our case. The top-performing ML model is the XGB with oversampled training data that achieved a BA of 0.742. However, it falls short of the performance achieved by the multimodal architecture, which reached a BA of 0.808, and NLP models employing data augmentation methods, which reached a BA of 0.815. 

These methods owe their high performance to different reasons, as mentioned before. NLP can learn how to extract information directly from the SMILES identifier without needing any predefined way to encode chemical information, so they have access to a higher concept of chemical representation without any pre-processing. The same is true for one of the branches of multimodal models, since it is based on the NLP approach, but can also integrate other sources of information encoded by other approaches such as MD, various fingerprints, and CDDD that fill the possible lack of purely chemical information derived from managing directly and only the SMILES as a string.

## 4. Discussion and Conclusions

We developed ML and AI models to assess the potential cardiotoxic effects of chemicals belonging to different classes such as pesticides, drugs, and industrializers. We followed linear AOP developed specifically for the cardiotoxicity endpoint, and this enabled us to select precise well-defined endpoints such as MIEs and KEs that describe possible molecular interactions between compounds and biological targets. Following the AOP theory is, from our point of view, one of the best approaches to selecting well-defined endpoints for modeling, which is fundamental in the regulatory perspective, as mentioned in OECD Environment Health and Safety Publications, Series on Testing and Assessment No. 69, Paris 2007 [33].

With our models, we evaluated the potential hazard of chemicals for inhibition of mitochondrial complexes, increase in oxidative stress [34,35], and increased mitochondrial dysfunction [36], and this makes this battery of models a promising first-tier component for new-approach methodologies (NAMs) or next-generation risk assessments (NGRAs) for cardiotoxicity. The use of in silico approaches in NAM-assisted toxicology, such as AI and ML models, to predict hazards is progressing fast, with the potential to transform the toxicology field by providing greater understanding of the mechanisms underlying chemical toxicity and permitting the development of safer and more sustainable products. 

We carried out different experiments to build advanced and high-performance AI networks, showing that these approaches outperform the baseline ML methods when the conditions are favorable, as in cases where the data run into the thousands. It is important to bear in mind that general ML models, due to their intrinsic properties, reach a plateau in performance that cannot be improved further even when new data become available. This is not true for AI approaches such as DNN or other architecture tested here. NLP methods and those that could manage different types of chemical encoders offer great promise, as we show here; the latter can learn from different chemical representations and further improve their ability to predict potential toxicity. Nevertheless, multimodal models are generally more complex due to the integration of multiple chemical encoders. Managing and optimizing such complexity can be challenging, both in terms of model architecture and training procedures. Integrating data from different encoders may pose challenges in terms of preprocessing and data elaboration. Additionally, combining information from multiple representations often requires more computational resources compared to unimodal models, leading to higher training times and resource requirements. The computational cost is a challenging problem not only during the training phases, as there are many cloud platforms available for performing calculations, but also for inference, reducing the models’ availability and practical use for the ultimate users. Other limitations of these methodologies may be linked to the fact that increasing the complexity of models often sacrifices model transparency. The challenge, therefore, lies in crafting models where each layer of complexity serves a purpose, rendering them both powerful and comprehensible. The ‘black box’ aspect associated with advanced AI methods leads the final user to prefer more transparent approaches, potentially sacrificing predictive capacity and performance. The need to increase the transparency of these approaches is often recognized, and indeed, a stronger emphasis on the model’s explainability is considered crucial. Various methods, including SHAP and LIME [31,32], appear to be promising solutions as well as integrating a self-attention mechanism into the network [37] with the goal of exploring the reasoning behind the models’ assessments and providing a chemical or toxicological explanation.

In the future, we hope to improve the model’s ability in different aspects. For instance, we want to develop multitask models to manage all the data provided for all possible events that may lead to cardiotoxic effects. This would allow our models to learn the potential toxicity of compounds in parallel on different MIEs and KEs. If possible, we would also like to include another mode of action such as hERG channel inhibition, which is a fundamental endpoint for evaluating cardiotoxicity, increasing the amount of data used by the models to learn the cardiotoxic effects. 

## Figures and Tables

**Table 1:** Assays to predict MIE and KEs for cardiotoxicity.

ASSAYS to Increase of Oxidative Stress	ASSAYS to Increase in Mitochondrial Dysfunction	ASSAYS to Inhibition of Mitochondrial Complexes
APR HepG2 P-H2AX 24 h dn	APR HepG2 MitoMass 24 h dn	CCTE Simmons MITO basal resp rate OCR dn
APR HepG2 P-H2AX 24 h up	APR HepG2 MitoMass 24 h up	CCTE Simmons MITO basal resp rate OCR up
APR HepG2 P-H2AX 72 h dn	APR HepG2 MitoMass 72 h dn	CCTE Simmons MITO inhib resp rate OCR dn
APR HepG2 P-H2AX 72 h up	APR HepG2 MitoMass 72 h up	CCTE Simmons MITO inhib resp rate OCR up
APR HepG2 StressKinase 24 h dn	APR HepG2 MitoMembPot 24 h dn	CCTE Simmons MITO max resp rate OCR dn
APR HepG2 StressKinase 24 h up	APR HepG2 MitoMembPot 24 h up	CCTE Simmons MITO max resp rate OCR up
APR HepG2 StressKinase 72 h dn	APR HepG2 MitoMembPot 72 h dn	
	APR HepG2 MitoMembPot 72 h up	
	ATG XTT Cytotoxicity up	
	TOX21 MMP ratio down	
	TOX21 MMP ratio up	
	TOX21 MMP rhodamine	

**Table 2:** Summary of data for each endpoint with information, number of compounds, and percentages of active and inactive compounds.

AOP	Name	Number of Compounds	Active	Inactive	Active%	Inactive%	Number of Assays Used
MIE1	Inhibition Mitochondrial Complexes	232	184	48	79	21	6
KE1	Increase Oxidative Stress	636	191	445	30	70	7
KE2	Mitochondrial Dysfunctions	5004	1147	3857	23	77	12

**Table 3:** Summary of data and splits for each endpoint, with information about the data and number of training and test compounds.

AOP	Name	Number of Compounds	Training Data	Test Data	SPLIT %
MIE1	Inhibition Mitochondrial Complexes	232	209	23	90–10
KE1	Increase Oxidative Stress	636	572	64	90–10
KE2	Mitochondrial Dysfunctions	5004	4504	500	90–10
KE2	Mitochondrial Dysfunctions	5004	4003	1001	80–20
KE2	Mitochondrial Dysfunctions	5004	3503	1501	70–30

**Table 4:** Results for the best ML models.

	External Test Set						Training Set CV Grid Optimization 5-Fold				
	Balance Accuracy	Precision	Sensitivity	Specificity	MCC	F1-Score	Balance Accuracy	Model Selected	Oversampling	Encoders	SPLIT%
*Inhibition of mitochondrial complexes*	0.721	0.889	0.842	0.600	0.415	0.865	0.833	*k-nearest neighbors*	SMOTE	*Mordred molecular descriptors*	90–10
*Increase in oxidative stress*	0.720	0.542	0.684	0.756	0.415	0.605	0.748	*Logistic regression*	SVM-SMOTE	*Mordred molecular descriptors*	90–10
*Increased mitochondrial dysfunction*	0.742	0.605	0.600	0.883	0.485	0.602	0.921	*Extreme gradient boosting*	SMOTE	*Latent description CDDD*	90–10
*Increased mitochondrial dysfunction*	0.747	0.615	0.607	0.887	0.500	0.611	0.922	*Extreme gradient boosting*	SMOTE	*Latent description CDDD*	80–20
*Increased mitochondrial dysfunction*	0.758	0.626	0.628	0.889	0.516	0.627	0.922	*Extreme gradient boosting*	SMOTE	*Latent description CDDD*	70–30

**Table 5:** Results of permutation importance method on KNN models for MIE1. The descriptors with the highest values are reported.

Weight	Feature
0.0194 ± 0.0178	MAXsOH
0.0164 ± 0.0030	MAXdssC
0.0139 ± 0.0082	GATS7d
0.0121 ± 0.0086	AATS5i
0.0115 ± 0.0045	AATS8se
0.0103 ± 0.0030	NssS

**Table 6:** Results for the AI models are reported for split 90–10. Each row represents a distinct AI architecture. The first of the three sections relates to the evaluation of the models on the external test set, the second assesses the model in training, and the third contains information on the encoder used.

	Test Set					Training Set		
	Balance Accuracy	Precision	Sensitivity	Specificity	MCC	F1-Score	Balance Accuracy	Encoder
*DNN Circular Fingerprint*	0.746	0.527	0.672	0.819	0.454	0.591	0.870	*Circular Fingerprint*
*DNN MACCS*	0.700	0.446	0.638	0.762	0.358	0.525	0.737	*MACCS fingerprint*
*DNN CDDD*	0.808	0.539	0.828	0.788	0.542	0.653	0.836	*Latent representation CDDD*
*DNN Molecular Descriptors*	0.774	0.471	0.828	0.720	0.470	0.600	0.811	*Molecular Descriptors*
*MPNN*	0.746	0.527	0.672	0.819	0.454	0.591	0.741	*Graph*
*NLP chars Embedding*	0.780	0.551	0.741	0.819	0.510	0.632	0.753	*Text Vectorization and character embedding*
***NLP chars Embedding Augmented***	**0.815**	**0.616**	0.776	0.855	**0.585**	**0.687**	0.886	***Text Vectorization and character embedding***
*Multimodal*	0.808	0.592	**0.777**	0.839	0.564	0.672	0.830	*All (no graph)*
*Extreme Gradient Boosting (best ML with oversampling methods)*	0.742	0.605	0.600	**0.883**	0.485	0.602	0.921	*Latent Description CDDD*

## Data Availability

The data used to develop the models can be found on the Alternative Cloud Platform [38]. Also, the models themselves will be available soon on the platform.

## Supplementary Materials

The following supporting information can be downloaded at: https://www.mdpi.com/article/10.3390/toxics12010087/s1, Section S1. Machine learning models and descriptor validation; Section S2: Deep learning models; Section S3: Results for split 80–20 and 70–30 KE2 and experimental summary; Section S4: Indices used to evaluate the models; Section S5: Data distribution comparison.
