# Supplementary material for: Artificial Intelligence and Machine Learning Methods to Evaluate Cardiotoxicity following the Adverse Outcome Pathway Frameworks
Source: Toxics. 2024 Jan 19;12(1):87. doi: 10.3390/toxics12010087 (PMC10820364; doi:10.3390/toxics12010087)
Supplement: Supplementary file 1 [file toxics-12-00087-s001.zip › toxics-2775799-supplementary.pdf]

toxics

MDPI

Supplementary Materials: Artificial intelligence and machine learning methods to evaluate cardiotoxicity following the Adverse outcome pathway frameworks

Edoardo Luca Viganò, Davide Ballabio and Alessandra Roncaglioni

Section S1: Machine Learning models and descriptors validation

1.1 MIE: Inhibition of mitochondrial complexes

Cross-validation for 10 splits

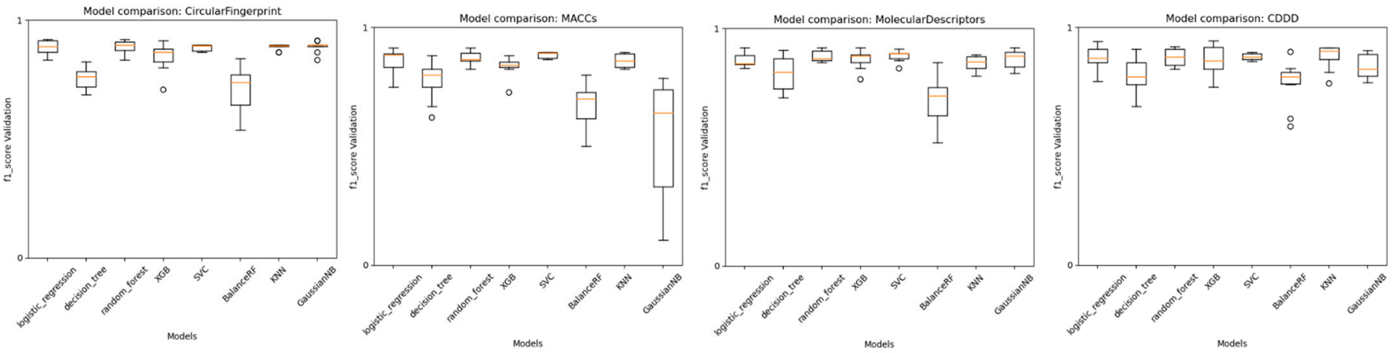

Figure S1: These box Plot figures represent a comparison, on 10-fold cross-validation, between different chemical descriptors, including Circular Fingerprint, MACCs, Mordred Molecular descriptors, and CDDD. The endpoint for the evaluation is the *Inhibition of mitochondrial complexes*. Different types of descriptors are used as input for various models reported in the title of each graph. The Median in cross-validation is reported in the orange row inside the boxplot.

The hyperparameter optimization was performed using grid search and the best model on the external test set is k-NN.

inhibition of mitochondrial complexes

Start calculation: kNN

Best hyperparameters: {'metric': 'manhattan', 'n_neighbors': 3, 'weights': 'distance'}

balanced_accuracy, 0.834

Total time taken: 0 minutes

Results External test set ------------------------------------

{'Balance Accuracy': 0.721, 'Precision': 0.889, 'Sensitivity': 0.842, 'Specificity': 0.6, 'MCC': 0.415, 'f1': 0.865}

Number of preds: 24

Active preds: 18

1.2 KE1: Increase Oxidative Stress

Cross-validation 10 split

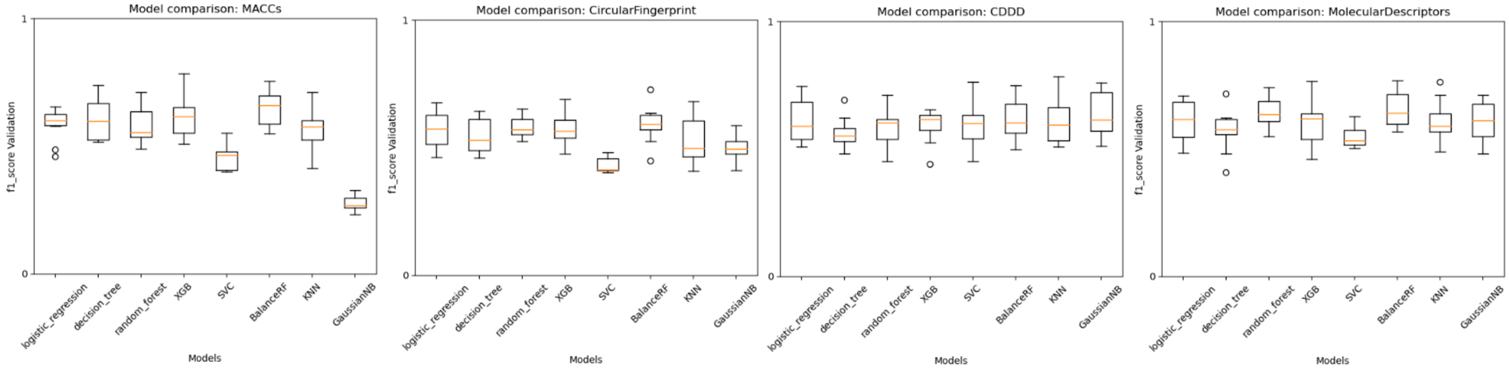

Figure S2: These box Plot figures represent a comparison, on 10-fold cross-validation, between different chemical descriptors, including Circular Fingerprint, MACCs, Mordred Molecular descriptors, and CDDD. The endpoint for the evaluation is the Increase Oxidative Stress Different types of descriptors are used as input for various models reported in the title of each graph. The Median in cross-validation is reported in the orange row inside the boxplot.

Cross-validation with Borderline-1 SMOTE oversampling using Molecular descriptors.

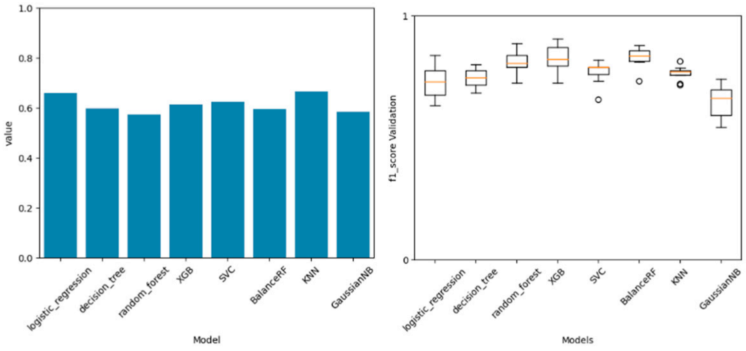

Figure S3: These figures represent results for (a) the cross-validation 10-fold and (b) the external test set. The types of ML models are reported on the x-axis. The training set used was oversampled in the minority class using SMOTE.
The hyperparameter optimization was performed using grid search and the best model on the external test set is Logistic Regression.

Increase in Oxidative stress

Start calculation: Logistic Regression

Best hyperparameters: {'C': 0.1, 'max_iter': 100, 'penalty': 'l2', 'solver': 'saga'}

balanced_accuracy, 0.723

Total time taken: 0 minutes

Results External test set -------------------------------------

{'Balance Accuracy': 0.664, 'Precision': 0.448, 'Sensitivity': 0.684, 'Specificity': 0.645, 'MCC': 0.302, 'f1': 0.542}

Number of preds: 64

Active preds: 29

1.3 KE2: Mitochondrial Dysfunction

Cross-validation 10 split

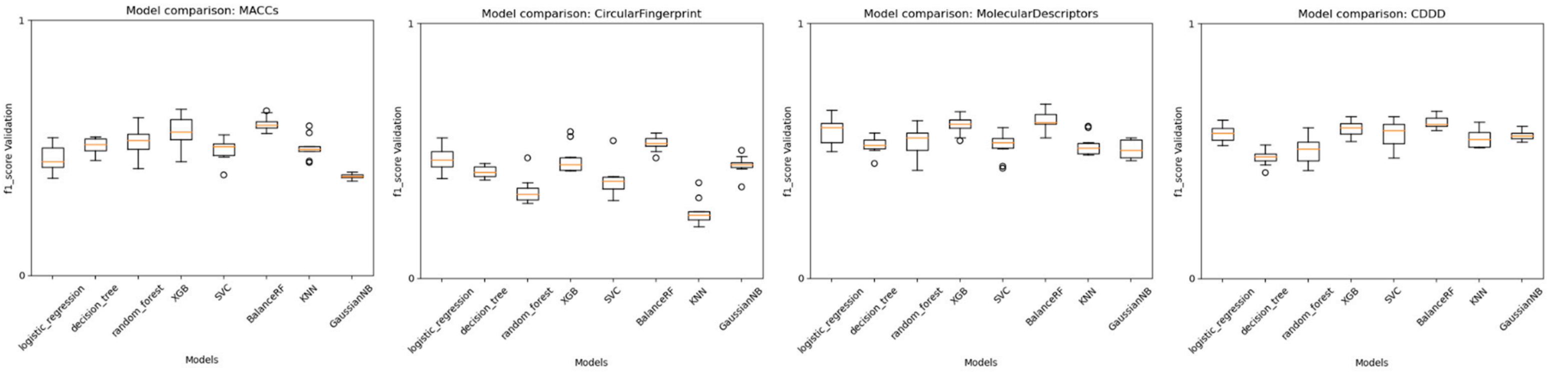

Figure S4: These box Plot figures represent a comparison, on 10-fold cross-validation, between different chemical descriptors, including Circular Fingerprint, MACCs, Mordred Molecular descriptors, and CDDD. The endpoint for the evaluation is *Mitochondrial Dysfunction*. Different types of descriptors are used as input for various models reported in the title of each graph. The Median in cross-validation is reported in the orange row inside the boxplot.

Cross-validation with SMOTE oversampling

To try to manage the unbalanced dataset, we test SMOTE oversampling the minority class for the best-performing descriptors the Mordred Molecular descriptors.

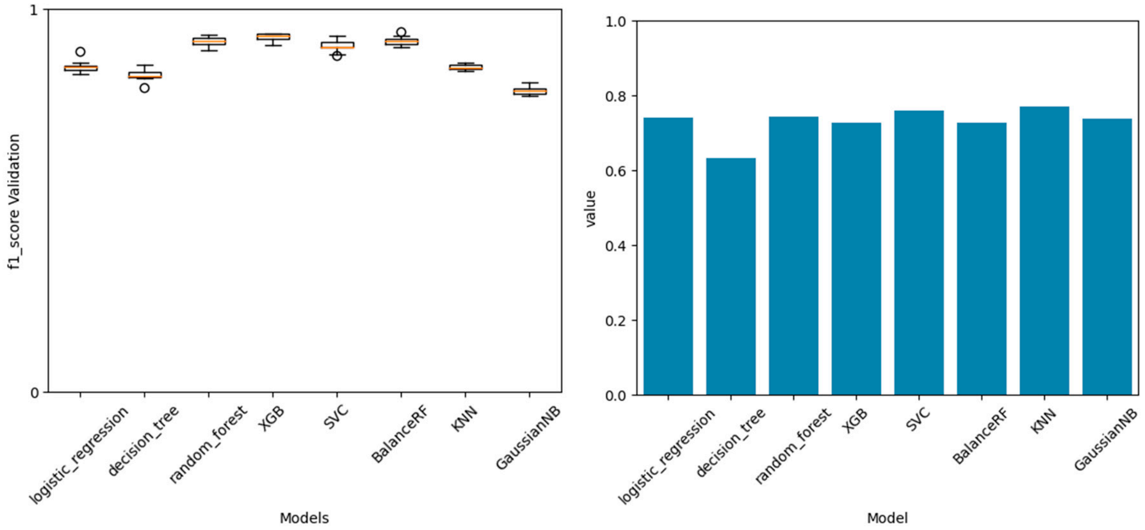

Figure S5: These figures show a comparison of different ML models evaluated on an external test set.

Increased Mitochondrial dysfunction

Start calculation: XGBclassifier

Best hyperparameters: {'colsample_bytree': 1.0, 'learning_rate': 0.5, 'max_depth': 5, 'n_estimators': 200, 'subsample': 1.0}

balanced_accuracy, 0.922

Total time taken: 216 minutes

Results External test set -------------------------------------

{'Balance Accuracy': 0.747, 'Precision': 0.615, 'Sensitivity': 0.610, 'Specificity': 0.887, 'MCC': 0.497, 'f1': 0.612}

Number of preds: 1001

Active preds: 226

Section S2: Deep Learning Models

2.1. Deep Neural Network

We built a simple Dense architecture to predict the mitochondrial toxicity on different chemical representations:

- Morgan circular fingerprint
- MACCs fingerprint
- CDDD descriptors
- Molecular Descriptors

The architecture is the same for each DNN, we choose the same for all descriptors to be able to better compare which of these chemical representations could be the best for our aims. The only difference is regarding the input layers whose dimensions depend on the features of the fingerprint, such as the *bin number*.

Layer (type)	Output Shape	Param #
FP_inputs (InputLayer)	[(None, shape_input)]	0
dense_16 (Dense)	(None, 232)	119016
batch_normalization_6 (BatchNormalization)	(None, 232)	928
dropout_10 (Dropout)	(None, 232)	0
batch_normalization_7 (BatchNormalization)	(None, 232)	928
dense_17 (Dense)	(None, 128)	29824
dropout_11 (Dropout)	(None, 128)	0
batch_normalization_8 (Batc	(None, 128)	512

```
hNormalization)

dense_18 (Dense)            (None, 64)            8256

dropout_12 (Dropout)        (None, 64)            0

dense_19 (Dense)            (None, 1)             65

=================================================================
Total params: 159,529
Trainable params: 158,345
Non-trainable params: 1,184
_________________________________________________________________
```

2.2 Neural Language Process

We have developed a model capable of predicting chemical toxicity directly from SMILES representations. It is possible to represent SMILES in a suitable format for layers typically used in NLP such as LSTM or GRU. For this purpose, we have to define the vocabulary of characters and perform text vectorization on the characters that compose SMILES.
This representation is used to create a character-level embedding where the model learns from data during optimization the best way to describe the character. For instance, figure S4 reported an example of embedding used in our models for calculating a specific molecule.

```
Charified text:
C N 1 C C N ( C ( = O ) O c 2 c 3 n c c n c 3 c ( O ) n 2 - c 2 c c c ( C l ) c n 2 ) C C 1

Length of random_train_chars: 46

Vectorized chars:
 [[ 2 10  7  2  2 10  5  2  5  8  6  4  6  3  9  3 12 11  3  3 11  3 12  3
   5  6  4 11  9 18  3  9  3  3  3  5  2 15  4  3 11  9  4  2  2  7  0  0
   0  0  0  0  0  0  0  0  0  0  0  0  0  0  0  0  0  0  0  0  0  0  0  0
   0  0  0  0  0  0  0  0  0  0  0  0  0  0  0  0  0  0  0  0  0  0  0  0
   0  0  0  0  0  0  0  0  0  0  0  0  0  0  0]]

Length of Vectorized chars: 111
Chiarified text:1n C N 1 C C N ( C ( = O ) O c 2 c 3 n c c n c 3 c ( O ) n 2 - c 2 c c c ( C l ) c n 2 ) C C 1

Embedding:
 [[[ 0.0025637   0.03526796 -0.02779629 ...  0.0060787   0.03064049
   -0.03925943]
  [-0.04418314  0.01262743 -0.00675542 ... -0.0131175  -0.03927482
    0.03514269]
  [-0.01162801 -0.03018752 -0.02386877 ...  0.03255202 -0.00092153
   -0.03525937]
  ...
  [ 0.02574723  0.03190776  0.00656862 ... -0.02340719 -0.02407622
   -0.0059273 ]
  [ 0.02574723  0.03190776  0.00656862 ... -0.02340719 -0.02407622
   -0.0059273 ]
  [ 0.02574723  0.03190776  0.00656862 ... -0.02340719 -0.02407622
   -0.0059273 ]]]

Character embedding shape: (1, 111, 264)
```

Figure S6: the image shows an example of the embedding process result done on SMILES.

Using this numerical representation an AI model with convolution and long-short-term memory (LSTM) bidirectional layers is trained.

Model architecture:

Layer (type)	Output Shape	Param #
inputs_smiles_as_char (InputLayer)	[(None, 1)]	0
char_vectorizer (TextVectorization)	(None, 111)	0
char_embed (Embedding)	(None, 111, 264)	7392
conv1d (Conv1D)	(None, 111, 128)	169088
conv1d_1 (Conv1D)	(None, 111, 64)	41024
bidirectional (Bidirectional)	(None, 111, 128)	66048
bidirectional_1 (Bidirectio	(None, 111, 64)	31104

```
 nal)

 global_max_pooling1d (Globa   (None, 64)           0
 lMaxPooling1D)

 dense (Dense)               (None, 64)            4160

 dropout (Dropout)            (None, 64)           0

 dense_1 (Dense)             (None, 32)            2080

 dropout_1 (Dropout)          (None, 32)           0

 dense_2 (Dense)             (None, 16)            528

 dense_3 (Dense)             (None, 1)             17

=================================================================
Total params: 321,441
Trainable params: 321,441
Non-trainable params: 0
```

2.3 Graph Neural Networks

Graph Neural Networks (GNNs) are a specialized type of neural network designed to learn from graph-structured data. In the context of chemistry, GNNs can be utilized to predict chemical properties by representing molecules as graphs. In this representation, each node in the graph corresponds to an atom, while each edge represents a chemical bond between the atoms. By leveraging GNNs, information can be efficiently propagated through the graph, effectively considering the relationships between the entities (atoms and bonds). The motivation for using this representation is because molecules are naturally represented as an undirected graph G = (V, E), where V is a set of vertices (nodes; atoms) and E a set of edges (bonds), GNNs (such as MPNN) are proving to be a useful method for predicting molecular properties.

This representation proves to be highly suitable for AI and ML applications, particularly due to the matrices that represent the graph's information, including the adjacency matrix, node features matrix, and bond features matrix. These matrices enable efficient processing and utilization of graph-based data, making GNNs a powerful tool for analyzing and predicting properties of chemical compounds.

One of the architectures we are going to use is a type of graph neural network (GNN) known as a message-passing neural network (MPNN) to predict graph properties.

GraphMMNN

Layer (type)	Output Shape	Param #	Connected to
atom_features (InputLayer)	[(None, 29)]	0	[]
bond_features (InputLayer)	[(None, 7)]	0	[]
pair_indices (InputLayer)	[(None, 2)]	0	[]
message_passing (MessagePassin g)	(None, 32)	14528	['atom_features[0][0]', 'bond_features[0][0]', 'pair_indices[0][0]']
molecule_indicator (InputLayer)	[(None,)]	0	[]
transformer_encoder_readout (T ransformerEncoderReadout)	(None, 32)	67008	['message_passing[0][0]', 'molecule_indicator[0][0]']
dense_26 (Dense)	(None, 512)	16896	['transformer_encoder_readout[0][0]']
dropout_16 (Dropout)	(None, 512)	0	['dense_26[0][0]']
dense_27 (Dense)	(None, 256)	131328	['dropout_16[0][0]']
dropout_17 (Dropout)	(None, 256)	0	['dense_27[0][0]']
dense_28 (Dense)	(None, 128)	32896	['dropout_17[0][0]']
dropout_18 (Dropout)	(None, 128)	0	['dense_28[0][0]']
dense_29 (Dense)	(None, 64)	8256	['dropout_18[0][0]']
dropout_19 (Dropout)	(None, 64)	0	['dense_29[0][0]']
dense_30 (Dense)	(None, 32)	2080	['dropout_19[0][0]']
dropout_20 (Dropout)	(None, 32)	0	['dense_30[0][0]']
dense_31 (Dense)	(None, 1)	33	['dropout_20[0][0]']

==

Total params: 273,025

Trainable params: 273,025

Non-trainable params: 0

2.4 Multimodal

Architecture Multimodal Neural Network

Layer (type)	Output Shape	Param #	Connected to
inputs_smiles_as_char (InputLayer)	[(None, 1)]	0	[]
char_vectorizer (TextVectorization)	(None, 111)	0	['inputs_smiles_as_char[0][0]']
char_embed (Embedding)	(None, 111, 264)	7392	['char_vectorizer[0][0]']
conv1d_2 (Conv1D)	(None, 111, 128)	169088	['char_embed[0][0]']
conv1d_3 (Conv1D)	(None, 111, 64)	41024	['conv1d_2[0][0]']
bidirectional_2 (Bidirectional)	(None, 111, 128)	66048	['conv1d_3[0][0]']
bidirectional_3 (Bidirectional)	(None, 111, 64)	31104	['bidirectional_2[0][0]']
global_max_pooling1d_1 (GlobalMaxPooling1D)	(None, 64)	0	['bidirectional_3[0][0]']
CFP_inputs (InputLayer)	[(None, 1024)]	0	[]
MACCs_inputs (InputLayer)	[(None, 167)]	0	[]
CDDD_inputs (InputLayer)	[(None, 512)]	0	[]
MD_inputs (InputLayer)	[(None, 399)]	0	[]
dense_19 (Dense)	(None, 64)	4160	['global_max_pooling1d_1[0][0]']

dense_22 (Dense)	(None, 128)	131200	['CFP_inputs[0][0]']
dense_25 (Dense)	(None, 128)	21504	['MACCs_inputs[0][0]']
dense_28 (Dense)	(None, 128)	65664	['CDDD_inputs[0][0]']
dense_31 (Dense)	(None, 128)	51200	['MD_inputs[0][0]']
batch_normalization_12 (BatchNormalization)	(None, 64)	256	['dense_19[0][0]']
batch_normalization_14 (BatchNormalization)	(None, 128)	512	['dense_22[0][0]']
batch_normalization_16 (BatchNormalization)	(None, 128)	512	['dense_25[0][0]']
batch_normalization_18 (BatchNormalization)	(None, 128)	512	['dense_28[0][0]']
batch_normalization_20 (BatchNormalization)	(None, 128)	512	['dense_31[0][0]']
dropout_12 (Dropout)	(None, 64)	0	['batch_normalization_12[0][0]']
dropout_14 (Dropout)	(None, 128)	0	['batch_normalization_14[0][0]']
dropout_16 (Dropout)	(None, 128)	0	['batch_normalization_16[0][0]']
dropout_18 (Dropout)	(None, 128)	0	['batch_normalization_18[0][0]']
dropout_20 (Dropout)	(None, 128)	0	['batch_normalization_20[0][0]']
dense_20 (Dense)	(None, 32)	2080	['dropout_12[0][0]']
dense_23 (Dense)	(None, 64)	8256	['dropout_14[0][0]']
dense_26 (Dense)	(None, 64)	8256	['dropout_16[0][0]']
dense_29 (Dense)	(None, 64)	8256	['dropout_18[0][0]']

dense_32 (Dense)	(None, 64)	8256	['dropout_20[0][0]']
batch_normalization_13 (BatchNormalization)	(None, 32)	128	['dense_20[0][0]']
batch_normalization_15 (BatchNormalization)	(None, 64)	256	['dense_23[0][0]']
batch_normalization_17 (BatchNormalization)	(None, 64)	256	['dense_26[0][0]']
batch_normalization_19 (BatchNormalization)	(None, 64)	256	['dense_29[0][0]']
batch_normalization_21 (BatchNormalization)	(None, 64)	256	['dense_32[0][0]']
dropout_13 (Dropout)	(None, 32)	0	['batch_normalization_13[0][0]']
dropout_15 (Dropout)	(None, 64)	0	['batch_normalization_15[0][0]']
dropout_17 (Dropout)	(None, 64)	0	['batch_normalization_17[0][0]']
dropout_19 (Dropout)	(None, 64)	0	['batch_normalization_19[0][0]']
dropout_21 (Dropout)	(None, 64)	0	['batch_normalization_21[0][0]']
dense_21 (Dense)	(None, 16)	528	['dropout_13[0][0]']
dense_24 (Dense)	(None, 64)	4160	['dropout_15[0][0]']
dense_27 (Dense)	(None, 64)	4160	['dropout_17[0][0]']
dense_30 (Dense)	(None, 64)	4160	['dropout_19[0][0]']
dense_33 (Dense)	(None, 64)	4160	['dropout_21[0][0]']
multimodal_inputs (Concatenate)	(None, 272)	0	['dense_21[0][0]', 'dense_24[0][0]', 'dense_27[0][0]', 'dense_30[0][0]', 'dense_33[0][0]']

dense_35 (Dense)	(None, 32)	8736	['multimodal_inputs[0][0]']
batch_normalization_23 (BatchNormalization)	(None, 32)	128	['dense_35[0][0]']
dropout_23 (Dropout)	(None, 32)	0	['batch_normalization_23[0][0]']
dense_36 (Dense)	(None, 16)	528	['dropout_23[0][0]']
dense_37 (Dense)	(None, 1)	17	['dense_36[0][0]']

==

Total params: 653,521

Trainable params: 651,729

Non-trainable params: 1,792

Section S3: Results for split 80 – 20 and 70 – 30 KE2 and experimental summary.

Table S1: split 80-20 modeling results.

	Test set						Training set	
	**Balance Accuracy**	**Precision**	**Sensitivity**	**Specificity**	**MCC**	**F1-Score**	**Balance Accuracy**	**Encoder**
DNN_CircularFingerprint	0.673	0.413	0.600	0.746	0.309	0.489	0.879	*Circular Fingerprint*
DNN_MACCs	0.734	0.459	0.722	0.746	0.410	0.561	0.755	*MACCs fingerprint*
DNN_CDDD	0.768	0.492	0.774	0.762	0.469	0.601	0.852	*Latent representation CDDD*
DNN_MolecularDescriptors	0.773	0.468	0.826	0.720	0.468	0.597	0.818	*Molecular Descriptors*
MPNN	0.726	0.518	0.626	0.826	0.425	0.567	0.749	*Graph*
NLP_charsEmbedding	0.712	0.532	0.574	0.850	0.413	0.552	0.736	*Text Vectorization and character embedding*
NLP_charsEmbeddingAugmented	0.733	0.586	0.591	0.876	0.466	0.589	0.792	*Text Vectorization and character embedding*
Multimodal	0.781	0.520	0.774	0.788	0.498	0.622	0.878	*All (no graph)*
Extreme Gradient Boosting (best ML with oversampling methods	0.747	0.615	0.607	0.887	0.500	0.611	0.922	*Latent Description CDDD*

Table S2: split 70-30 modeling results.

	Test set						Training set	
	**Balance Accuracy**	**Precision**	**Sensitivity**	**Specificity**	**MCC**	**F1-Score**	**Balance Accuracy**	**Encoder**
DNN_CircularFingerprint	0.725	0.525	0.616	0.834	0.427	0.567	0.880	*Circular Fingerprint*
DNN_MACCs	0.736	0.438	0.762	0.710	0.405	0.556	0.756	*MACCs fingerprint*
DNN_CDDD	0.796	0.564	0.767	0.824	0.536	0.650	0.846	*Latent representation CDDD*
DNN_MolecularDescriptors	0.789	0.511	0.808	0.770	0.506	0.626	0.817	*Molecular Descriptors*
MPNN	0.760	0.543	0.692	0.827	0.480	0.609	0.736	*Graph*
NLP_charsEmbedding	0.762	0.538	0.703	0.820	0.481	0.610	0.822	*Text Vectorization and character embedding*
NLP_charsEmbeddingAugmented	0.759	0.555	0.680	0.838	0.484	0.611	0.900	*Text Vectorization and character embedding*
Multimodal	0.743	0.496	0.698	0.789	0.438	0.580	0.858	*All (no graph)*
Extreme Gradient Boosting (best ML with oversampling methods	0.758	0.626	0.628	0.889	0.516	0.627	0.922	*Latent Description CDDD*

Table S3: Experimental summary

				SPLIT %			*OVERSAMPLING*			
	Dataset	***Encoders***	***Models***	**70-30**	**80-20**	**90-10**	*SMOTE*	*BORDERLINE SMOTE*	*SVMSMOTE*	ADASYN
Machine Learning	***Inhibition Mitochondrial complexes, Increase Oxidative Stress** and **Increased Mitochondrial dysfunction***	Circular Fingerprint	Logistic Regression	KE2	KE2	all endpoints				
			Decision Tree	KE2	KE2	all endpoints				
			Random Forest	KE2	KE2	all endpoints				
			Extreme Gradient Boosting	KE2	KE2	all endpoints				
			Support Vector Machine	KE2	KE2	all endpoints				
			Balance Random Forest	KE2	KE2	all endpoints				
			k-nearest neighbors	KE2	KE2	all endpoints				
			Gaussian Naive Bayes	KE2	KE2	all endpoints				
		MACCs fingerprint	Logistic Regression	KE2	KE2	all endpoints				
			Decision Tree	KE2	KE2	all endpoints				
			Random Forest	KE2	KE2	all endpoints				
			Extreme Gradient Boosting	KE2	KE2	all endpoints				
			Support Vector Machine	KE2	KE2	all endpoints				
			Balance Random Forest	KE2	KE2	all endpoints				
			k-nearest neighbors	KE2	KE2	all endpoints				
			Gaussian Naive Bayes	KE2	KE2	all endpoints				
		Molecular Descriptors	Logistic Regression	KE2	KE2	all endpoints	x	x	x	x
			Decision Tree	KE2	KE2	all endpoints	x			
			Random Forest	KE2	KE2	all endpoints	x			
			Extreme Gradient Boosting	KE2	KE2	all endpoints	x			
			Support Vector Machine	KE2	KE2	all endpoints	x			
			Balance Random Forest	KE2	KE2	all endpoints	x			
			k-nearest neighbors	KE2	KE2	all endpoints	x	x	x	x
			Gaussian Naive Bayes	KE2	KE2	all endpoints	x			
		Latent representation CDDD	Logistic Regression	KE2	KE2	all endpoints				
			Decision Tree	KE2	KE2	all endpoints				
			Random Forest	KE2	KE2	all endpoints				
			Extreme Gradient Boosting	KE2	KE2	all endpoints				
			Support Vector Machine	KE2	KE2	all endpoints				
			Balance Random Forest	KE2	KE2	all endpoints				
			k-nearest neighbors	KE2	KE2	all endpoints				
			Gaussian Naive Bayes	KE2	KE2	all endpoints				
Atificial Intelligence Deep Neural Networks	***Increased Mitochondrial dysfunction***	Circular Fingerprint	Deep Neural Network	KE2	KE2	KE2				
		MACCs fingerprint	Deep Neural Network	KE2	KE2	KE2				
		Molecular Descriptors	Deep Neural Network	KE2	KE2	KE2				
		Graph	Message Passing Neural Networks	KE2	KE2	KE2				
		charsEmbedding	Neural Lenguage Processing	KE2	KE2	KE2				
		charsEmbeddingAugmented	Neural Lenguage Processing	KE2	KE2	KE2				
		Latent representation CDDD	Deep Neural Network	KE2	KE2	KE2				
		All(no graph)	Multimodal	KE2	KE2	KE2				

Section S4: Indices used to evaluate the models.

- Precision:

$$Precision = \frac{True\ Positive}{True\ Positive + False\ Positive}$$

- Sensitivity:

$$Sensitivity = \frac{True\ Positive}{True\ Positive + False\ Negative}$$

- Specificity:

$$Specificity = \frac{True\ Negative}{True\ Negative + False\ Positive}$$

- Balance Accuracy:

$$Balance\ Accuracy = \frac{Sensitivity + Specificity}{2}$$

- F1-score: is the harmonic mean of the precision and recall. It thus symmetrically represents both precision and recall in one metric.

$$F1 - score = 2 * \frac{Precision * Recall}{Precision + Recall}$$

- Accuracy: shows how often a classification ML model is correct overall

$$Accuracy = \frac{True\ Positive + True\ Negative}{True\ Positive + False\ Positive + True\ Negative + False\ Negative}$$

- Matthew Correlation Coefficient:
  True Positive = TP
  True Negative = TN
  False Positive = FP
  False Negative = FN

$$MCC = \frac{(TP * \mathrm{TN}) * (\mathrm{FP} * \mathrm{FN})}{\sqrt{(TP + FP) * (TP + FN) * (TN + FP) * (TN + FN)}}$$

Section S5: Data distribution comparison

MIE: Inhibition of mitochondrial complexes

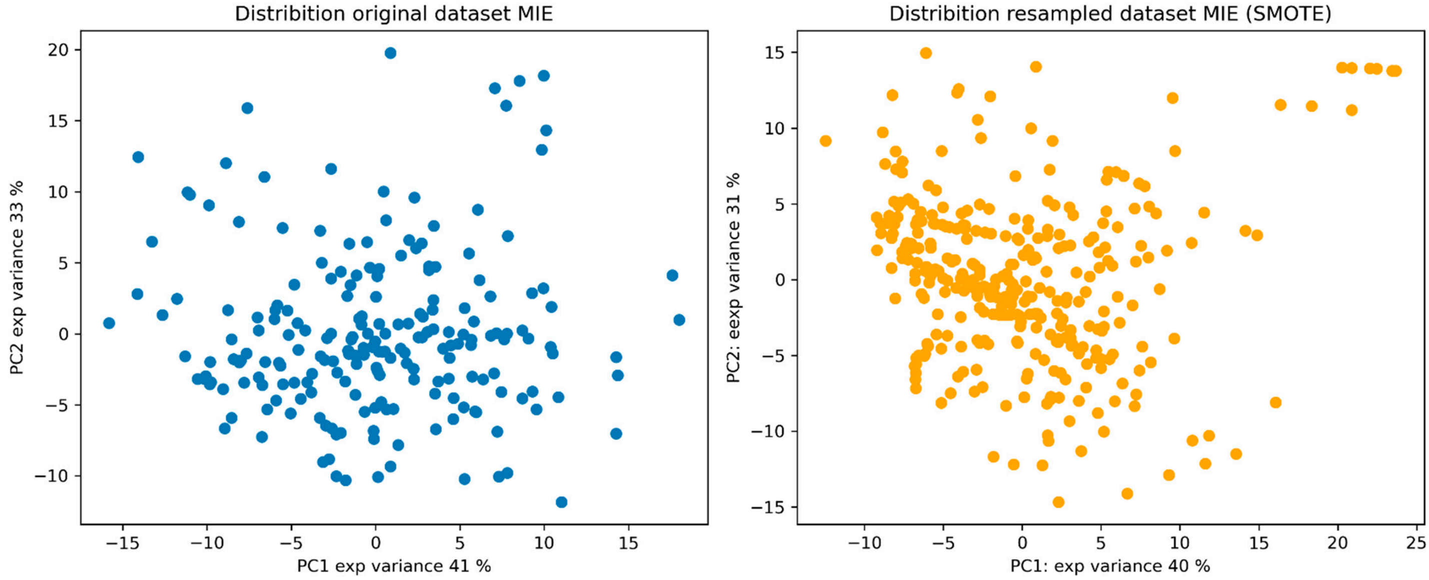

Figure S7: A comparison of the original data distribution for the "Inhibition of Mitochondrial Complexes" endpoint and the data generated by SMOTE methods. The data are plotted in principal component space.

PC1

Kolmogorov-Smirnov Statistic: 0.083

P-value: 0.316

No significant difference observed.

KE1: Increase Oxidative Stress

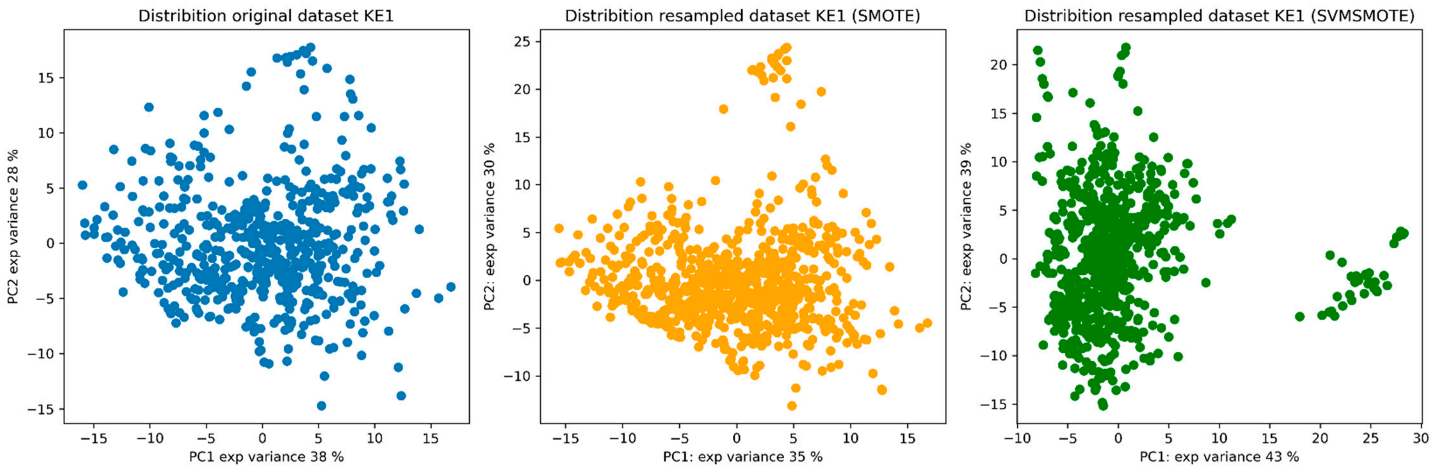

Figure S8: A comparison of the original data distribution for the " Increase Oxidative Stress " endpoint and the data generated by SMOTE and SVMSMOTEmethods. The data are plotted in principal component space.

PC1:

SMOTE---

Kolmogorov-Smirnov Statistic: 0.0313

P-value: 0.885 No significant difference observed.

SVMSMOTE---

Kolmogorov-Smirnov Statistic: 0.247

P-value: 2.10e-18 The distributions are significantly different.

KE2: Mitochondrial Dysfunction

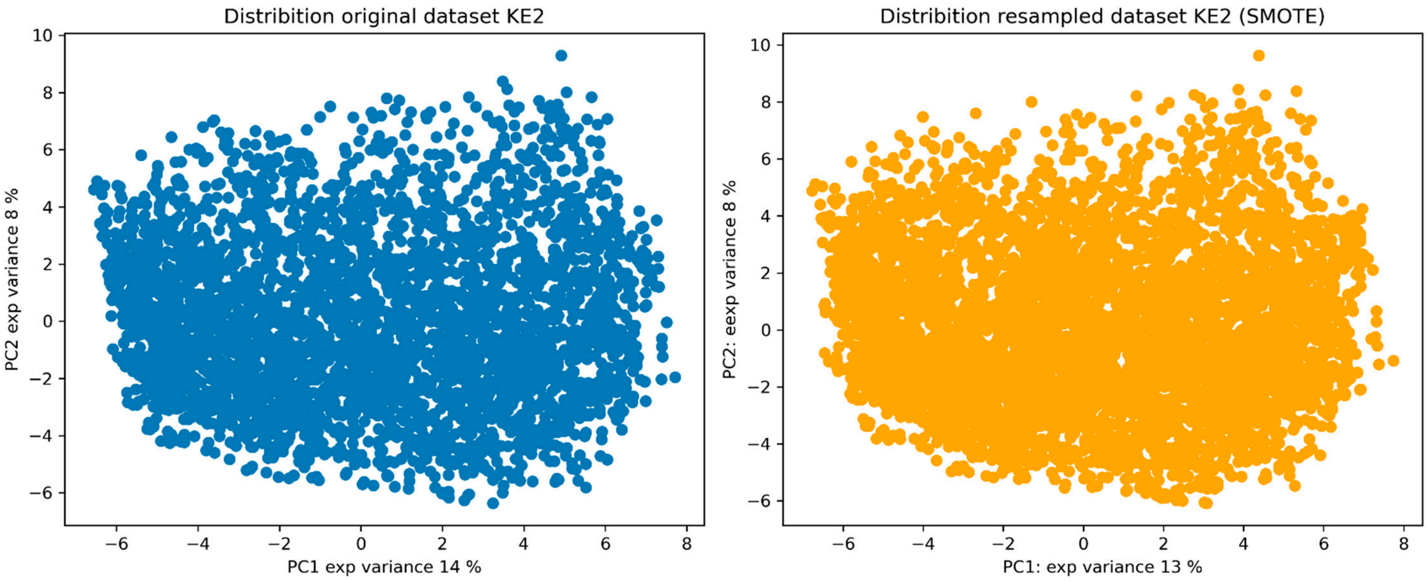

Figure S9: A comparison of the original data distribution for the " Mitochondrial Dysfunction" endpoint and the data generated by SMOTE methods. The data are plotted in principal component space.

PC1

Kolmogorov-Smirnov Statistic: 0.0234

P-value: 0.136

No significant difference observed.
